# Supplementary material for: Opposing microtubule motors control motility, morphology and cargo segregation during ER-to-Golgi transport
Source: Biol Open. 2014 Apr 4;3(5):307–13. doi: 10.1242/bio.20147633 (PMC4021352; doi:10.1242/bio.20147633)
Supplement: Supplementary Material [file supp_3_5_307_v2_index.html]

Opposing microtubule motors control motility, morphology and cargo segregation during ER-to-Golgi transport — Supplementary Material 

# Opposing microtubule motors control motility, morphology and cargo segregation during ER-to-Golgi transport

## bio.20147633 Supplementary Material

**Files in this Data Supplement:**

- Supplementary Material - Anna K. Brown et al. doi: 10.1242/bio.20147633
