# Supplementary material for: Opposing microtubule motors control motility, morphology and cargo segregation during ER-to-Golgi transport
Source: Biol Open. 2014 Apr 4;3(5):307–13. doi: 10.1242/bio.20147633 (PMC4021352; doi:10.1242/bio.20147633)
Supplement: Supplementary Material [file supp_bio.20147633_bio.20147633-s1.pdf]

Biology Open (2014) 000, 1–7 doi:10.1242/bio.20147633

Supplementary Material

Anna K. Brown et al. doi: 10.1242/bio.20147633

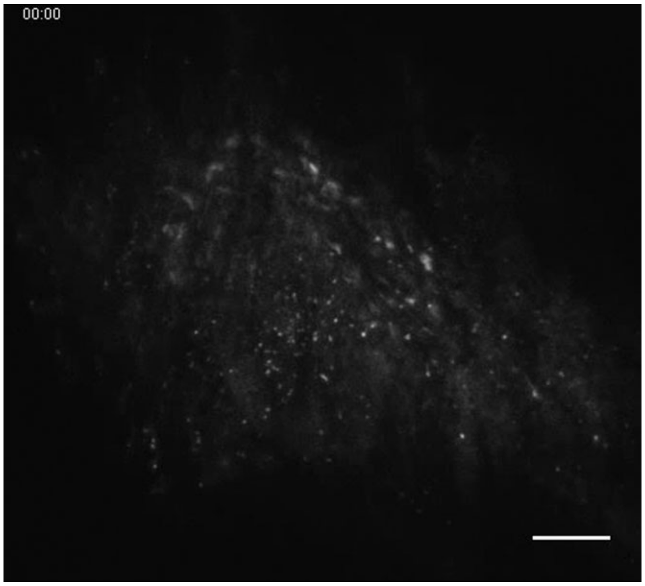

Movie 1A. GL2 cells transfected with siRNA duplexes (playback at real time). TIRF imaging of tsO45-G-GFP 12 minutes after temperature shift of cells to 32˚C. See Fig. 1A. Movie hosted at this site: http://dx.doi.org/10.5523/bris.14s2rmstmx6am1k8jkcd3g9uz3

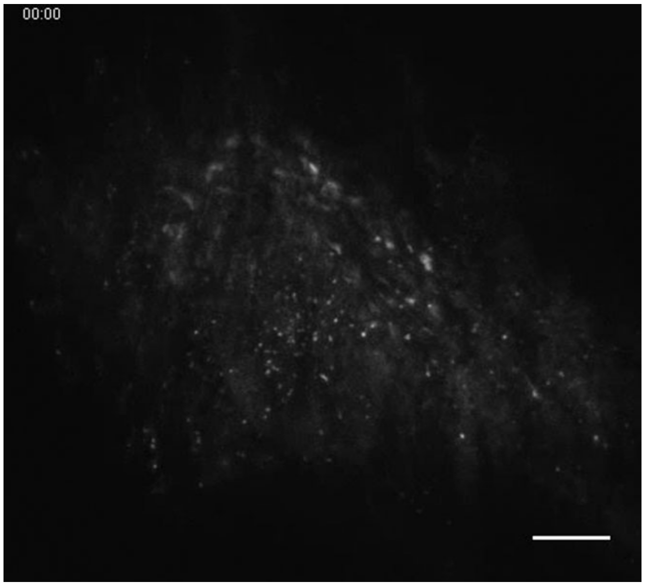

Movie 1B. GL2 cells transfected with siRNA duplexes (playback at 3× real time). TIRF imaging of tsO45-G-GFP 12 minutes after temperature shift of cells to 32˚C. See Fig. 1A. Movie hosted at this site: http://dx.doi.org/10.5523/bris.14s2rmstmx6am1k8jkcd3g9uz3

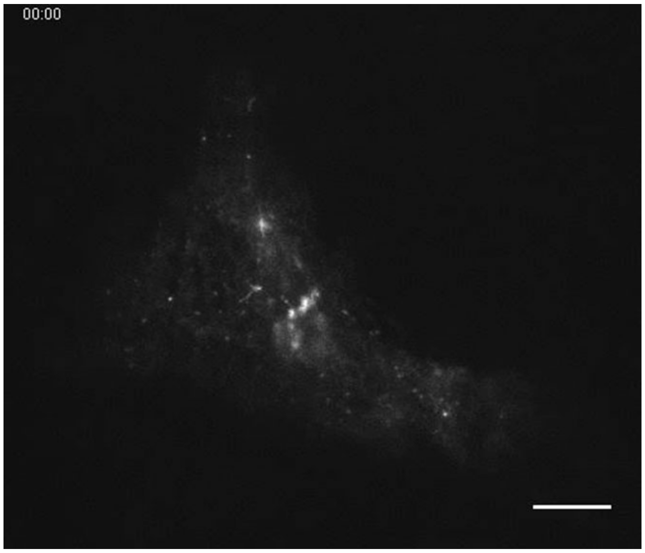

Movie 2A. DHC1 cells transfected with siRNA duplexes (playback at real time). TIRF imaging of tsO45-G-GFP 12 minutes after temperature shift of cells to 32˚C. See Fig. 1D. Movie hosted at this site: http://dx.doi.org/10.5523/bris.14s2rmstmx6am1k8jkcd3g9uz3

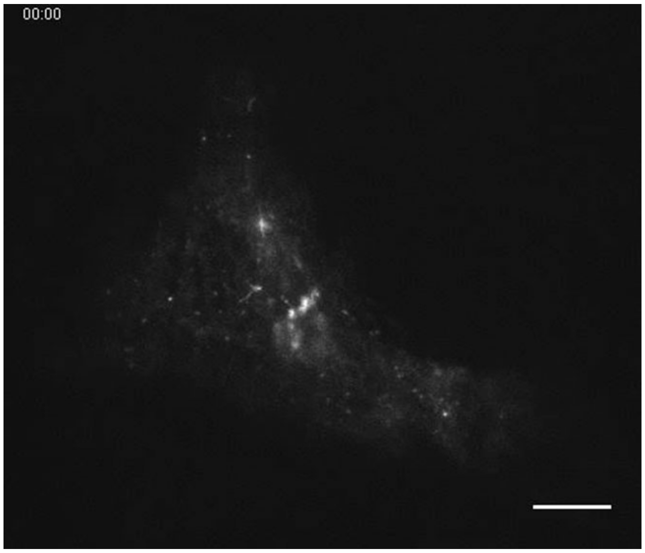

Movie 2B. DHC1 cells transfected with siRNA duplexes (playback at 3× real time). TIRF imaging of tsO45-G-GFP 12 minutes after temperature shift of cells to 32˚C. See Fig. 1D. Movie hosted at this site: http://dx.doi.org/10.5523/bris.14s2rmstmx6am1k8jkcd3g9uz3

Biology Open

Biology Open (2014) 000, 1–7 doi:10.1242/bio.20147633

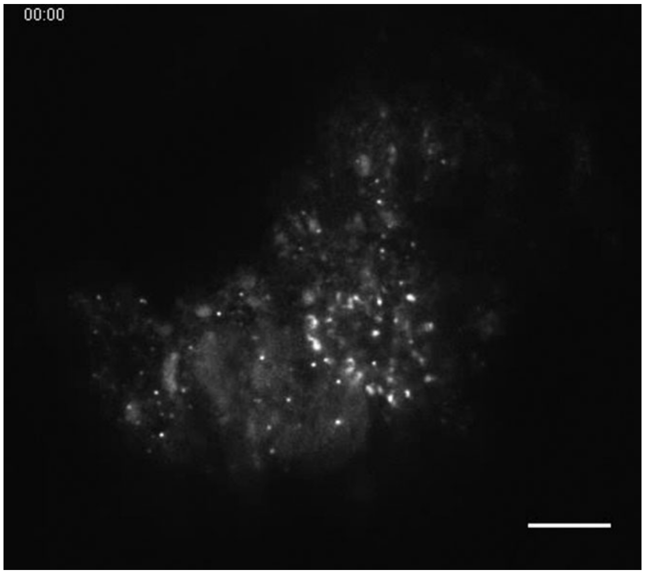

Movie 3A. LIC1 cells transfected with siRNA duplexes (playback at real time). TIRF imaging of tsO45-G-GFP 12 minutes after temperature shift of cells to 32˚C. See Fig. 1G. Movie hosted at this site: http://dx.doi.org/10.5523/bris.14s2rmstmx6am1k8jkcd3g9uz3

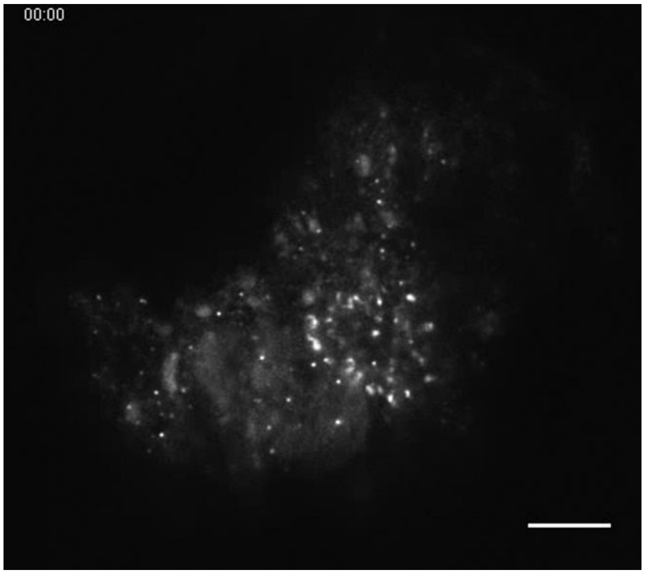

Movie 3B. LIC1 cells transfected with siRNA duplexes (playback at 3× real time). TIRF imaging of tsO45-G-GFP 12 minutes after temperature shift of cells to 32˚C. See Fig. 1G. Movie hosted at this site: http://dx.doi.org/10.5523/bris.14s2rmstmx6am1k8jkcd3g9uz3

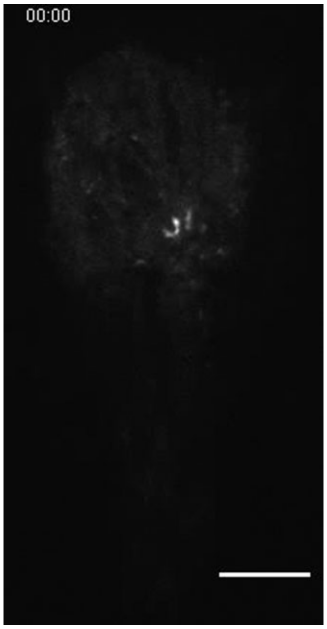

Movie 4A. KIF5B cells transfected with siRNA duplexes (playback at real time). TIRF imaging of tsO45-G-GFP 12 minutes after temperature shift of cells to 32˚C. See Fig. 1J. Movie hosted at this site: http://dx.doi.org/10.5523/bris.14s2rmstmx6am1k8jkcd3g9uz3

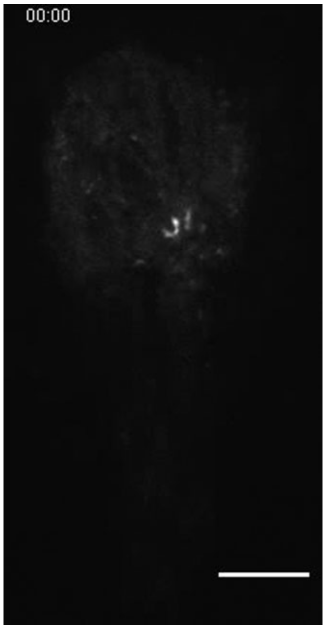

Movie 4B. KIF5B cells transfected with siRNA duplexes (playback at 3× real time). TIRF imaging of tsO45-G-GFP 12 minutes after temperature shift of cells to 32˚C. See Fig. 1J. Movie hosted at this site: http://dx.doi.org/10.5523/bris.14s2rmstmx6am1k8jkcd3g9uz3

Biology Open

Biology Open (2014) 000, 1–7 doi:10.1242/bio.20147633

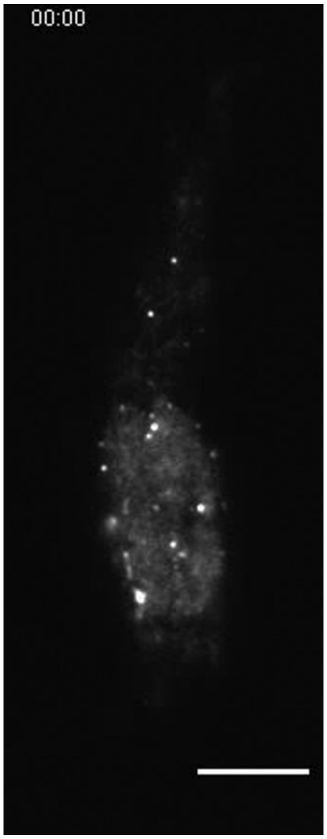

Movie 5A. KAP3 cells transfected with siRNA duplexes (playback at real time). TIRF imaging of tsO45-G-GFP 12 minutes after temperature shift of cells to 32˚C. See Fig. 1M. Movie hosted at this site: http://dx.doi.org/10.5523/bris.14s2rmstmx6am1k8jkcd3g9uz3

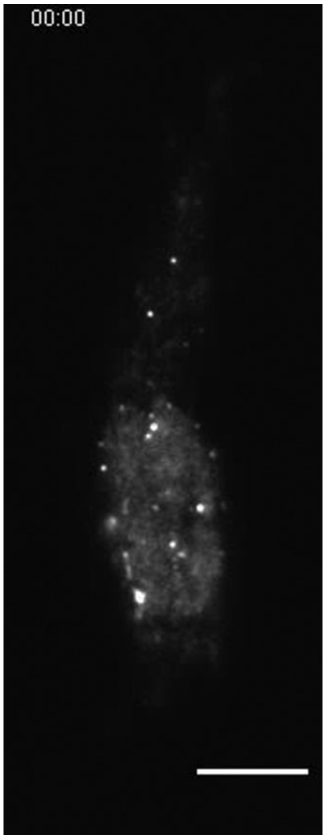

Movie 5B. KAP3 cells transfected with siRNA duplexes (playback at 3× real time). TIRF imaging of tsO45-G-GFP 12 minutes after temperature shift of cells to 32˚C. See Fig. 1M. Movie hosted at this site: http://dx.doi.org/10.5523/bris.14s2rmstmx6am1k8jkcd3g9uz3

Biology Open
